# University of Venda’s male students’ attitudes towards contraception and family planning

**DOI:** 10.4102/phcfm.v8i2.959

**Published:** 2016-07-08

**Authors:** Nanga R. Raselekoane, Keamogetse G. Morwe, Takalani Tshitangano

**Affiliations:** 1 Institute for Gender and Youth Studies, University of Venda, South Africa; 2 Department of Public Health, University of Venda, South Africa

## Abstract

**Background:**

Many young men continue to disregard the importance of contraception and family planning in South Africa. The fact that even university students also do not take contraception and family planning seriously poses a serious threat to their own health and well-being.

**Aim:**

This paper aims at investigating the attitudes of male students towards contraception and the promotion of female students’ sexual health rights and well-being at the University of Venda.

**Methods:**

Quantitative research method is used to determine how attitudes of 60 male students towards contraception can jeopardise the health and well-being of both male and female students.

**Results:**

This study reveals that the majority of 60 male students at the University of Venda have a negative attitude towards contraceptives. As a result, male students at the University of Venda are not keen on using contraceptives. Male students’ negative attitude and lack of interest in contraceptives and family planning also limit progress in achieving the Millennium Development Goals on primary health care, especially with regard to sexual and reproductive health and well-being of female students at the University of Venda.

**Conclusion:**

The fact that more than half of the male students interviewed did not take contraception and family planning seriously poses a serious threat to health and well-being of students, including violation of female students’ sexual and reproductive health rights in South Africa. This calls for radical health promotion and sexual and reproductive rights programmes which should specifically target male students at the University of Venda.

## Introduction

Despite the flood of information on contraception in South Africa, the rate of unintended pregnancies, HIV and/or AIDS, sexually-transmitted infections (STIs), abortion-related morbidity and mortality among teenagers continues to rise as a result of engagement in unprotected sex by most young people.^1,2,3,4,5,6,7,8,9,10^ South Africa has the highest number of people living with HIV and/or AIDS as it accounts for an estimated 5.4 million (25.0%) cases of the HIV infections in sub-Saharan Africa.^1^

The developing countries carry the largest number of unintended HIV infections, pregnancies, abortions and maternal mortality in the world. For example, Ethiopia accounts for 440 deaths per 100 000 births and 70 000 abortion-related mortality and morbidity every year among young women.^7,11,12^ These high levels of HIV infections, abortions, abortion-related morbidity and maternal deaths can be prevented by changing young men’s attitudes towards contraception and family planning, especially in developing countries where the challenges associated with the non-use of contraceptives are endemic. Otherwise, the loss of life of many young women from non-use of contraceptives will never be curbed.^13^

In South Africa, more students in tertiary institutions show limited use of contraceptives. As a result, most of them are resorting to emergency contraception.^8,14^ The fact that only 37.0% of the unmarried sexually active young women aged 15–24 years in sub-Saharan Africa use contraception is extremely worrying, especially in view of the devastating effects of unsafe sex.^11^ Religion and culture have always been used to oppose the use of contraceptives. But now cultural norms have shifted and teenage pregnancy is not seen as immoral anymore.^13,15^ However, today poverty also plays a major role in pushing the health authorities to promote the use of contraceptives since many people have no access to quality health care.^16,17^ There is a need to scale up the agenda of urging more young men to use contraceptives.^18,19,20,21,22^

There are multiple barriers to the use of contraceptives by young people. Most parents see contraception and family planning as sensitive issues that cannot be discussed with children. The community also has a negative attitude towards sexually active unmarried young people. There is a general belief that people who use contraceptives are promiscuous. Such a belief is partly responsible for the non-use of contraceptives among the youth.^15,23^ Increased use of contraceptives in South Africa is warranted by the high rates of HIV and/or AIDS infections and the ever-increasing AIDS-related deaths of young people. The well-being of young people is extremely important, and it needs to be given priority at homes and educational institutions. While there is a dramatic increase of contraceptive use in Asia and Latin America, there is still a serious challenge in sub-Saharan Africa, especially among young men. The use of contraceptives in Africa is still below 20.0%, with only 6.0% of the married men using condoms.^24^

It is extremely worrying that in this day and age most of the men in South Africa are not using contraception, as compared with 56.0% of South African women who use contraceptives.^25^ This, probably, accounts for the highest unintended pregnancy and abortion rates in South Africa.^26,27^ The limited or inconsistent use of contraceptives among tertiary students in South Africa also poses a serious threat to young people’s health and well-being. Most young people have misconceptions about the safety of contraceptives because of inadequate and incorrect information.^14,28^ For example, in Nigeria, most of the refugee youths (49.0%) believe that contraceptives are dangerous and damaging to their health and well-being. These myths about contraception often make young people, especially young men, resistant to contraception. If young people’s health and well-being have to be protected, these societal myths about contraception among young people warrant an urgent attention.^26,29,30,31,32,33^

Male students in particular have a negative attitude towards contraception. They believe that contraceptives decrease sexual pleasure, are unreliable and affect their sexual spontaneity. This shows that people generally view contraception as a hindrance to good health, although this is not true.^28,29,33,34,35,36,37^ This also shows that young people do not see the importance of using contraceptives, hence, high levels of teenage pregnancy, HIV and/or AIDS infections and STIs in South Africa. The distribution of inaccurate information about contraception and reproductive health is extremely dangerous as it is driving many young people not to use contraceptives, especially among less-educated people.^8,36,38,39^

In traditional African settings, a man’s opinion is dominant and women cannot independently decide about using contraceptives. They need permission from their husbands or boyfriends to do so. This means that women are often forced against their will not to use contraceptives. In South Africa some boyfriends reportedly even use violence to stop their girlfriends from using contraceptives. As a result, their clinic cards may be torn apart by their boyfriends and pills thrown away. Some young men even demand that their girlfriends fall pregnant to prove their fertility. The fact that men are physically strong and also that they generate income make them feel entitled to dictating to their female partners not to use contraceptives. Some young men use religious beliefs as an excuse to prevent their girlfriends from using contraceptives.^36,38,40,41,42^ Sociocultural beliefs also inhibit young people from using contraceptives. In male-dominated societies, women are discouraged from using contraceptives. Men often use culture to subjugate their partners and prevent them from using contraceptives.^18,28,29,37,43^

Spousal influence, gender power relations, religious beliefs and sociocultural factors collaborate in making it difficult for young men to use contraceptives. As a result, their health is compromised. Such a situation also leaves most young women vulnerable as they can easily fall pregnant or contract sexually transmitted diseases. This works against the realisation of the Millennium Development Goals on promotion of gender equality and women empowerment, improvement of maternal health and combating HIV and/or AIDS.

### Purpose of study

The purpose of this study is to investigate male students’ attitudes towards contraception and the promotion of female students’ sexual health rights and well-being at the University of Venda.

### Objectives

The objective of the study is to determine contributory factors to the attitudes of the University of Venda’s male students towards contraception and family planning.

## Research methods and design

### Study design

This study is quantitative in nature. It gives a broader explanation and understanding of the knowledge and attitudes of male students towards contraceptives. The descriptive survey research design, a subtype of the non-experimental research design, has been used to collect data on the knowledge and attitudes of male students at the University of Venda towards contraception and family planning.^44,45^

### Study population and sampling strategy

The study was conducted at the University of Venda located in Thohoyandou in the Limpopo Province (South Africa). The study population consisted of 450 registered University of Venda male students aged between 18 and 24 years. The sample comprised 60 students. Stratified random sampling, a subtype of probability sampling,^29,44,45,46^ was used to select male students for this study. The participants were divided into four groups, each with 15 students. This was to ensure proportional representation of male students in the 1st, 2nd, 3rd and 4th years.

### Data collection

The research questionnaire and 4 focus groups with 15 participants each were used to collect data. The research questionnaire was pre-tested on 4 male students with the same characteristics as those in the sample. This was done to validate the questionnaire’s validity, reliability and appropriateness to the study. Vague and difficult questions were refined following pilot testing. The questionnaires were self-administered randomly around the University of Venda campus after the aim of the research was explained to the respondents.

### Data analysis

The collected data were analysed through the use of the SPSS tool.^45,46^ The data were assigned codes for the purpose of analysis and interpretation of data.

### Trustworthiness

The object, scope and setting of the study have been clearly explained. Relevant research methods and sample which are appropriate for the study have been selected and used in this study. Quantitative research approach and stratified random sampling as well as a structured questionnaire and focus groups were used to collect data on the knowledge and attitudes of male students towards contraception and family planning.^47^ The use of structured questionnaire and the focus group complement each other and also helped to ensure that all questions adequately covered and were answered.

### Ethical considerations

Approval to conduct this study was obtained from the Directorate of Research and Innovation at the University of Venda. To avoid abuse and harm for research participants, the researcher informed the research participants about the importance of informed consent, confidentiality, anonymity and voluntary participation.^44,46^

## Results

See Table 1 for a presentation of the study on the knowledge and attitudes of male students towards contraception and family planning at the University of Venda.

**TABLE 1:** Knowledge and awareness of contraceptives by male students.

Variable	Yes (%)	No (%)
Awareness of contraceptives	55 (92.0)	5 (8.0)
Awareness that contraceptives are used to prevent pregnancy	58 (98.0)	2 (2.0)

*Source*: Authors’ own work

About 92% and 98% of interviewed male students have, respectively, shown a high level of awareness about contraceptives and use of contraceptives to prevent pregnancy. Only 8% and 2% of the respondents, respectively, stated that they were not aware of contraceptives or different types of contraceptives and the use of contraceptives for the prevention of pregnancy.

**FIGURE 1:**
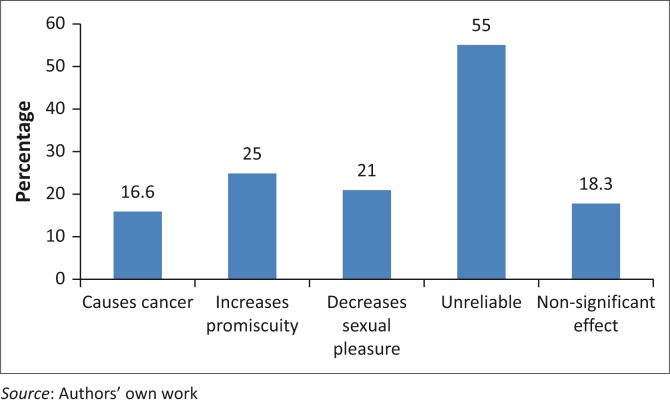
Negative attitudes towards contraception and family planning.

Figure 1 shows responses provided by the participants when they were responding to the question – *Why do you not like using contraceptives?*

Most of the interviewed male students at the University of Venda stated that they had a negative attitude towards contraception and family planning because contraceptives were unreliable (55.0%), cause cancer (16.6%), decrease sexual pleasure (25.0%), increase promiscuity (21.0%) and that they have no significant effect. Male students who said that contraceptives were unreliable constituted 18.3% of the sample.

According to Table 2, 58.3% (*n* = 26) of the interviewed male students were using contraceptives. Around 74.3% of the participants (*n* = 35) were using contraceptives to avoid impregnating their girlfriends, while 25.7% (*n* = 9) of the participants were using contraceptives to avoid STIs. About 41.7% (*n* = 25) of the respondents indicated that they were not using any method of contraception. About 48.0% (*n* = 12) of the participants indicated that they were not using contraceptives because they were not sexually active. Male students who were not using contraceptives because of the desire to impregnate their partners constituted 4.0% (*n* = 1). Those who were not using contraceptives because they were afraid of the side effects constituted 24.0% (*n* = 6). There were participants who were also not using contraceptives because of their partner’s insistence (4% [*n* = 1]) and religious influence (16% [*n* = 4]), respectively.

**TABLE 2:** Experiences of male students with contraception.

Variable	*N*	%
Participants using contraceptives	35	58.3
Use of contraceptives to prevent pregnancy	26	74.3
Use of contraceptives to prevent sexually-transmitted infections	9	25.7
Non-use of contraceptives	25	41.7
Non-use of contraceptives because of sexual inactivity	12	48.0
Non-use of contraceptives because of the intention to fall pregnant	1	4.0
Non-use of contraceptives because of fear of the side effects	6	24.0
Non-use of contraceptives because of partner’s influence	1	4.0
Non-use of contraceptives because of religious influence	4	16.0

*Source*: Authors’ own work

In Table 3, the majority of the participants (i.e., 88.3%) indicated that contraceptives were not always reliable. However, 16.7% indicated that contraceptives were always reliable. More than 66.7% of the respondents agreed that it was good to use contraceptives while 30.0% of the respondents indicated the contrary. More than 55.0% of the interviewed male students did not like to use contraceptives and only 35.0% liked to use contraceptives.

**TABLE 3:** Attitudes of male students towards contraception.

Variable	Answer	%
Contraceptives always reliable	Yes No	83.3 16.7
Is it good to use contraceptives?	Yes No	66.7 30.0
Like to use contraceptives	Yes No	35.0 55.0

*Source*: Authors’ own work

In Table 4, the majority of male students (91.7%) have indicated their awareness of different types of contraceptives as opposed to only 8.3% who did not know the different types of contraceptives.

**TABLE 4:** Male students’ awareness of the different types of contraceptives.

Variable	Number (%)
Aware of different types of contraceptives	55 (91.7)
Not aware of different types of contraceptives	5 (8.3)

*Source*: Authors’ own work

Table 5 shows that the majority of the respondents (95%) knew the condom as the only method to prevent STIs. Only 1.7% of the respondents knew about the diaphragm as a method that could prevent STIs. About 3.3% of the participants did not know of any method which could prevent STIs.

**TABLE 5:** Knowledge of contraceptives that can prevent sexually-transmitted infections.

Variable	%
Condom	95.0
Do not know any	5.0

*Source*: Authors’ own work

Some of the interviewed male students (36.7% [*n* = 22]) indicated that culture acts as barrier to the use of contraceptives whereas only 28.3% (*n* = 17) stated that culture did not act as a barrier to contraception. About 35% (*n* = 21) did not know if culture acted as a barrier to the use of contraceptives. More than half of the interviewed male students (53.3%, *n* = 32) said that their community did not support the use of contraceptives, whereas 18.3% (*n* = 11) said that their community did not bar them from using contraceptives. About 26.7% (*n* = 16) of the participants did not know if their society supported the use of contraception (see Table 6).

**TABLE 6:** Cultural, societal and religious barriers to contraception.

Variable	Yes (%)	No (%)	Do not know (%)
Culture support the use of contraceptives	22 (36.7)	17 (28.3)	21 (35)
Society act as a barrier to contraception	32 (53.3)	11 (18.3)	16 (26.7)

*Source*: Authors’ own work

According to 36.7% (*n* = 22) of the participants, religion did not support the use of contraception. On the contrary, 31.7% (*n* = 19) of the participants indicated that their religion was not a barrier to contraception. About 28.3% (*n* = 17) of the interviewed male students did not know if their religion supported the use or non-use of contraceptives.

Table 7 shows that 60.3% (*n* = 41) of the participants had discussed the issue of contraception with their partners, whereas 33.7% (*n* = 19) had never discussed contraception with their partners.

**TABLE 7:** Discussion with partners about contraception.

Variable	Number (%)
Discussed with partners about contraception	36 (60.3)
Not discussed with partners about contraception	19 (33.7)

*Source*: Authors’ own work

## Discussion

The study reveals a high level of awareness of contraceptives (91.7%) among University of Venda’s male students. This is similar to the case of college students in the United States who were also aware of the availability of contraceptives.^31^ Most of the respondents (96.7%) indicated that they knew that contraceptives were used to prevent pregnancy. Only 3.3% of the respondents said they were not aware that contraceptives were used to prevent pregnancy.

Most of the participants (65.0%) indicated that they first became aware of contraceptives at the secondary level, followed by 23.3% at the primary level and 8.3% at the tertiary level. This is similar to the study conducted by Ankitage^33^ in which most of the respondents (71.2%) knew about family planning during their secondary school level of education, 15.0% at the primary level and 13.1% at the tertiary level.

Although most male students showed a high level of awareness about contraception, only 38.0% of them knew the correct way of using contraceptives. This is almost similar to a study conducted in India where the level of awareness was 41.1% but the actual knowledge regarding contraception was 7.3%.^32^ About 83.3% of the participants indicated that there were some benefits associated with contraception, such as pregnancy prevention (65.0%) and the prevention of STIs (27.0%).^33,48^ Only a negligible 8.0% of the respondents maintained that contraception had no significant benefit for them.

It is worrying that despite a high level of awareness or knowledge of contraceptives, the majority of the male students (55.0%) at the University of Venda displayed a negative attitude towards contraception and family planning for different reasons. The fact that 58.0%, 16.6%, 21.0%, 25.0%, 18.3% of the participants, respectively, indicated that contraceptives were unreliable, cause cancer, decrease sexual pleasure and also increase promiscuity may make them not to use contraceptives.

According to Table 2, 58.3% of the participants were currently using contraception. Male students using contraceptives in order to prevent pregnancy constituted 74.3% (*n* = 26). However, 25.7% (*n* = 9) of the respondents were only using contraceptives to prevent STIs while 41.7% (*n* = 25) of the respondents were not using any method of contraception. Also, 48% of the male students (*n* = 12) indicated that they were not using contraceptives because they were not sexually active. Male students who were not using contraceptives because of the desire to impregnate their partners constituted 4.0% (*n* = 1) of the interviewed respondents. Meanwhile, 24% (*n* = 6) of the participants stated that they were not using contraceptives because they were afraid of the side effects. About 4.0% (*n* = 1) and 16.0% (*n* = 4) of the male students stated that they were not using contraceptives because of their partner’s insistence and religious influence, respectively.

The data revealed that culture and religion played a pivotal role in shaping the attitudes of male students towards contraception and family planning. More than 73.4%, that is, 36.7% (*n* = 22) and 36.7% (*n* = 22) of male students, respectively, saw culture and religion as deterrents to the use of contraceptives. This means that these male students did not see it as an obligation on their part to take contraception as important and necessary. Therefore, the negative attitude of male students towards contraception caused by their culture and religion might have a negative effect on their sensitivity to their female partners’ sexual and reproductive health rights. Such a situation may have far reaching implications on the health and well-being of both male and female students at the University of Venda.

The fact that there were about 48.0% male students who were not using contraceptives is of great concern. This may compromise the health and well-being of the male students and their partners. Since the patriarchal ideology still underpins the thinking and practices as well as behaviour of most South African citizens, male students are likely to push their way to have unprotected sex with their female partners. This ideology gives male students the latitude to go ahead with their reckless behaviour of not using contraceptives. Such an act is likely to expose them and their partners to HIV and AIDS and STIs.

The study also shows that most male students’ negative attitude towards contraception and family planning was because of their belief that contraception causes all sorts of maladies such as cancer (16.0%), increased promiscuity (21.0%), decreased sexual pleasure and also that it is unreliable.^28^ Over 36.7% of the participants did not support the use of contraception because of their religious beliefs. There were also participants (28.3%) who indicated that their religion did not serve as an impediment in using contraceptives.

The study shows that more than half (60.3%) of the participants discussed the issue of contraceptives with their partners while 32.0% never did so. This might probably be because of the fear of rejection.^33^ The data further indicated that more than 75.0% of the partners of the participants supported the use of contraceptives while 21.7% were against that. Women’s experiences could also affect the way they could use contraceptives because some husbands and boyfriends do not support the use of contraceptives by their partners.^36^

This study also shows that male students were involved or engaged in the discussion of contraception and family planning. According to Table 7, more than half of the participants (60.0%, *n* = 36) had discussed the issue of contraception with their partners. Around 31.7% (*n* = 19) of the respondents had never discussed contraception with their partners. The latter respondents dictated to their partners not to use contraceptives as they alleged that contraception was harmful for them.

### Recommendations

Based on the main finding that male students at the University of Venda have no interest in contraception and family planning, the following recommendations are proposed to address this matter.

More trained sexual educators should be used to equip young men with life skills which will help them make informed decisions and also to take responsibility concerning contraception. Such skills would educate young men on how to protect themselves and their partners from HIV and unwanted pregnancies.

There is a greater need to provide male students with information on the effects of contraceptives. Sexual and reproductive health education should be included in the students’ curriculum so that they can be more aware of the challenges associated with unprotected sex.

Campus Health Clinic should intensify and implement robust awareness campaigns on health and well-being of students. Special programmes on the benefits of contraception and family planning specifically for male students on campus should be initiated in collaboration with student leadership and Campus Health Clinic. More special campaigns should be organised to sensitise male students to the needs and sexual and health rights of female students on campus. Ministries of Health, Social Development and Higher Education and Training should collaborate and intensify the campaigns on sexual and reproductive health rights in South Africa.

Collaboration involving university authorities, student leadership and Campus Health Clinic should be intensified and sustained to change male students’ mindset on contraception and family planning. The realisation of such an effort will be helpful in the quest for the attainment of Millennium Development Goals on primary health care, particularly the promotion and support for women’s sexual and reproductive health rights.

Traditional and religious systems also need to be overhauled to end male domination in a society that perpetuates subjugation of women and the subsequent undermining of their sexual and reproductive health and well-being.

## Conclusion

Most male students at the University of Venda do not take contraception and family planning seriously. As a result, male students do not see it as their responsibility to participate in contraception and family planning campaigns taking place on campus. They fail to realise that practising safe sex is the best and effective method of preventing pregnancy, HIV and STIs, as well as reducing pregnancy-related morbidity and mortality. Male students’ negative attitude towards contraception endangers their health and well-being as well as that of their partners.

Poor or low level of education, gender inequality, religious beliefs and sociocultural factors are just some of the factors that are responsible for male students’ negative attitude towards contraception and family planning. As long as this situation persists, male students at the University of Venda will continue to be reckless by engaging in unprotected sex. They will in turn endanger themselves and the female students on campus. The consequences of such a high-risk sexual behaviour by male students at the University of Venda are too ghastly to contemplate.

In view of the negative attitudes of male students towards contraceptives, the Campus Health Clinic at the University of Venda needs to urgently come up with a robust strategy to respond to this to protect the health and well-being of both male and female students. The Campus Health Clinic needs to intensify and implement radical health and well-being awareness campaigns specifically targeted at male students. Strong collaboration between university authorities, student leadership and Campus Health Clinic as well as Ministries of Health, Social Development and Higher Education and Training should be used to develop a multipronged strategy that can be used to address the attitudes of male students towards contraception and family planning. In other words, only concerted efforts involving different stakeholders within and outside the university can be helpful in crafting an intervention model that will help to change the mindset of male students on contraception and family planning.
